# Directed Evolution of Silicatein Reveals Biomineralization Synergism between Protein Sequences

**DOI:** 10.1021/acsomega.4c06359

**Published:** 2025-01-06

**Authors:** Toriana N. Vigil, Mary-Jean C. Rowson, Abigail J. Frost, Abigail R. Janiga, Bryan W. Berger

**Affiliations:** † Department of Chemical Engineering, University of Virginia, Charlottesville, Virginia 22903, United States; ‡ Department of Biomedical Engineering, University of Virginia, Charlottesville, Virginia 22903, United States

## Abstract

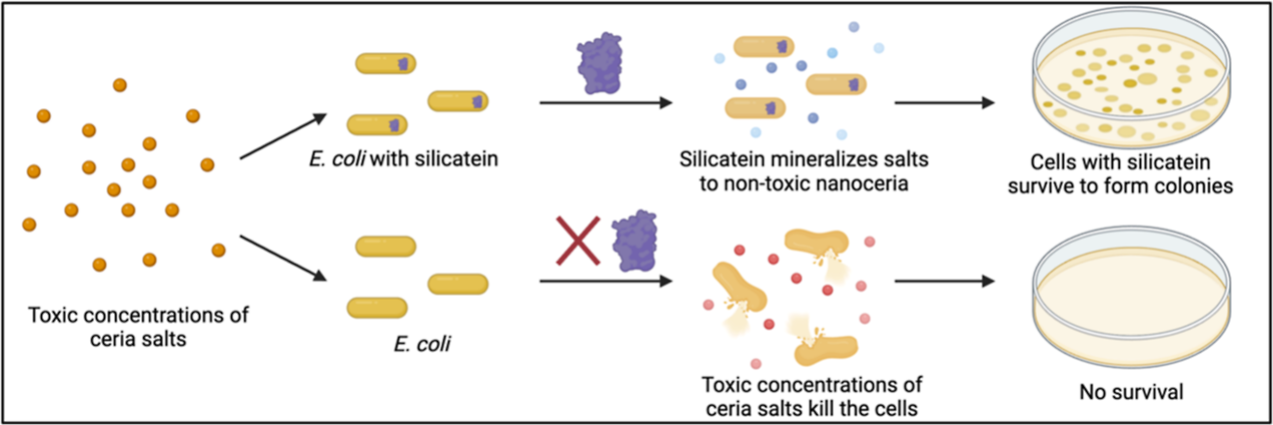

Biomineralization is a green synthesis route for a variety of metal nanoparticles. Silicatein is a biomineralization protein originally found in marine sponge *Tethya aurantia* that converts inorganic precursors to metal oxide nanoparticles. In this work, we investigate the popular catalytic triad hypothesis and implement directed evolution with the aim to improve the solubility and kinetics of silicatein to enable increased nanoparticle synthesis. Site-directed mutagenesis with catalytic triad residues did not abolish biomineralization activity, aligning with the results seen in one previous study. Recombinant production of silicatein and mutants in *Escherichia coli* following library generation and a survival screen yielded several mutant proteins with augmented biomineralization activity. Sequence analysis of these mutant proteins reveals multiple sequences within a single cell that contribute to enhanced biomineralization. Combined with the sequence analysis of silicateins from different marine sponges, these results suggest the protein is permissive to wide sequence variations and that multiple protein sequences act synergistically for enhanced biomineralization.

## Introduction

With an ever-increasing dependence on technology, our society has an increasing need for advanced materials, such as inorganic nanoparticles, which are generally made with wet chemistry techniques. Cerium oxide, or nanoceria, is of particular interest for applications in catalysis and medicine.^1−7^ Although wet chemistry synthesis techniques are established, they include the use of toxic reactants, organic solvents, and high temperatures, while producing toxic byproducts and often polydisperse particles.^8^ A green approach to nanoparticle synthesis harnesses natural biomineralization proteins that function under mild temperatures and in aqueous conditions.^9,10^

Silicatein is a biomineralization protein that produces monodisperse crystalline particles and is promising for materials synthesis applications.^11−15^ Silicatein was first discovered in marine sponge *Tethya aurantia*, forming the silica spicules integral to the sponge exoskeleton.^16^ Since then, analogous silicateins have been identified in other marine sponge species *Suberites domuncula*,^17^*Petrosia ficiformis*,^18^*Latrunculia oparinae*,^19^*Halichondria okadai*,^20^*Amphimedon queenslandica*,^21^ and even in freshwater sponge *Latrunculia baicalensis*.^22^ Notably, there is a significant genetic variation across these species, and genetic engineering approaches to transform diatoms for recombinant protein production often lead to mosaic colonies.^23^ In nature, silicatein has three separate subunits: silicatein alpha, silicatein beta, and silicatein gamma, of which silicatein alpha has been identified as capable of biomineralization activity independent from other subunits^16^ and henceforth been the subject of many studies.

The phylogenetic relationships of these proteins and genes was examined in 2007 by Müller et al., highlighting the evolution of silicateins in Porifera and the similarities with the Cathepsin family.^24^ The sequence similarity with cathepsin proteases contributes to the “catalytic triad hypothesis”, in which three amino acids (S26, H165, and N185) are postulated to be responsible for catalytic activity. The catalytic triad hypothesis is supported by early studies utilizing site-directed mutagenesis and evaluating biomineralization with silica.^24−27^ However, a 2018 study by Povarova et al. also performs site-directed mutagenesis with the catalytic triad residues and shows no significant differences in silica biomineralization activity.^28^

Many studies have also shown that although silicatein’s native substrate is silica, it also mineralizes titania, gallium, barium, cerium, and other metals.^11,29^ Through bioengineering and directed evolution, silicatein could become a valuable tool for nanoparticle synthesis with a variety of elements. Although silicatein is promising in these ways, the silicatein mineralization yields are significantly impacted by enzyme solubility and kinetics. A significant body of previous works examines different solubility tags to elicit a greater protein expression and solubility.^26,27,30^ The trigger factor silicatein fusion (TF-sil) significantly increases solubility and does not interfere with biomineralization activity. A recent work shows that extrinsic modifications to the protein, such as solubility tags, do not significantly impact biomineralization activity.^31^ Therefore, this work seeks to elicit intrinsic changes to the protein via a directed evolution approach in order to enhance the silicatein activity.

In 2012, Bawazer et al. sought a similar outcome via DNA shuffling and in vitro protein expression, utilizing silicatein alpha and silicatein beta as shuffling partners.^32^ In their work, Bawazer et al. examined mineralization with silica and titania and sampled 30 total mutants, identifying only two full-length proteins. In these full-length proteins, the catalytic triad was conserved. These findings supported the catalytic triad hypothesis, originally suggested by Zhou 1999, which proposes catalytic activity based on sequence similarity to cysteine protease Cathepsin L.^25^ Although many studies have examined this putative catalytic triad (S26, H165, and N185), the role of the catalytic triad in the mechanism of biomineralization remains an area of open debate.^25,27,28,33^

The uncertainty surrounding silicatein’s mechanism of action may stem in part from examining biomineralization with regard to silica. Although silica species are the native substrate for silicatein, in vitro silica biomineralization is consistently very low (ng produced/μg input).^26−28,31,34^ Furthermore, the popular precursor tetra-ethyl orthosilicate has significant autohydrolysis activity,^35^ resulting in relatively high background mineralization. Here, we evaluate biomineralization with precursor ceric ammonium nitrate (CAN), with which our lab has previously shown greater silicatein mineralization (mg produced/mg input),^31^ thus enabling a more robust comparison of biomineralization activity.

Our results with directed evolution of recombinant silicatein, we show that silicatein has notable sequence flexibility and that several silicatein mutants confer greater biomineralization than a single silicatein alone. Furthermore, this work suggests that enhanced biomineralization may be achieved best through the combination of several versions of silicatein in combination with one another, as seen with genetic mosaicism in a multitude of marine species.

## Results and Discussion

The TF-silicatein fusion protein has been shown to be the easiest to express and the most soluble of recombinant silicatein fusions.^26,27^ In order to limit confounding overall insolubility with activity, we used TF-silicatein with hexa-histidine tags (where the silicatein sequence is the synthetic construct derived from *S. domuncula* reported by Müller et al.^36^) as the wild type (WT) protein, focusing our site-directed mutagenesis and library around silicatein alone without making changes to the TF fusion partner. The sequence for WT TF-silicatein is shown in the Materials and Methods section. One report suggests that the hexa-histidine tag may be a major contributor to silicatein biomineralization activity with silica species.^26^ To control for the possibility that the hexa-histidine tag contributes to biomineralization activity, we performed a biomineralization experiment with hexa-histidine-tagged TF-silicatein and hexa-histidine-tagged Cathepsin L with substrate CAN. A two-tailed unpaired *t*-test shows that biomineralization yields of TF-silicatein are significantly greater than those with Cathepsin L (*p* < 0.005), thus illustrating that biomineralization activity is not solely attributable to the hexa-histidine tag and is consistent with TF-silicatein enzyme activity (Figure S2).

### Evaluating the Catalytic Triad Hypothesis with Ceria Biomineralization

Because there is significant uncertainty surrounding the catalytic triad hypothesis and little else is known about silicatein’s mechanism of action, we first evaluated the catalytic triad via site-directed mutagenesis and biomineralization with CAN. Because silicatein has previously shown greater biomineralization activity with ceria than with silica, evaluating activity with this substrate facilitates a more robust comparison. In this work, we quantified ceria biomineralization with an arsenazo III colorimetric assay, adapted from Hogendoorn et al.^37^ Furthermore, TF-silicatein activity tends to be relatively variable between different protein productions, so in order to standardize biomineralization activity, overall treatment of biomineralization yield data was performed according to the methods of Povarova et al. 2018.^28^ A full explanation is included in the Supporting Information.

**Figure 1:**
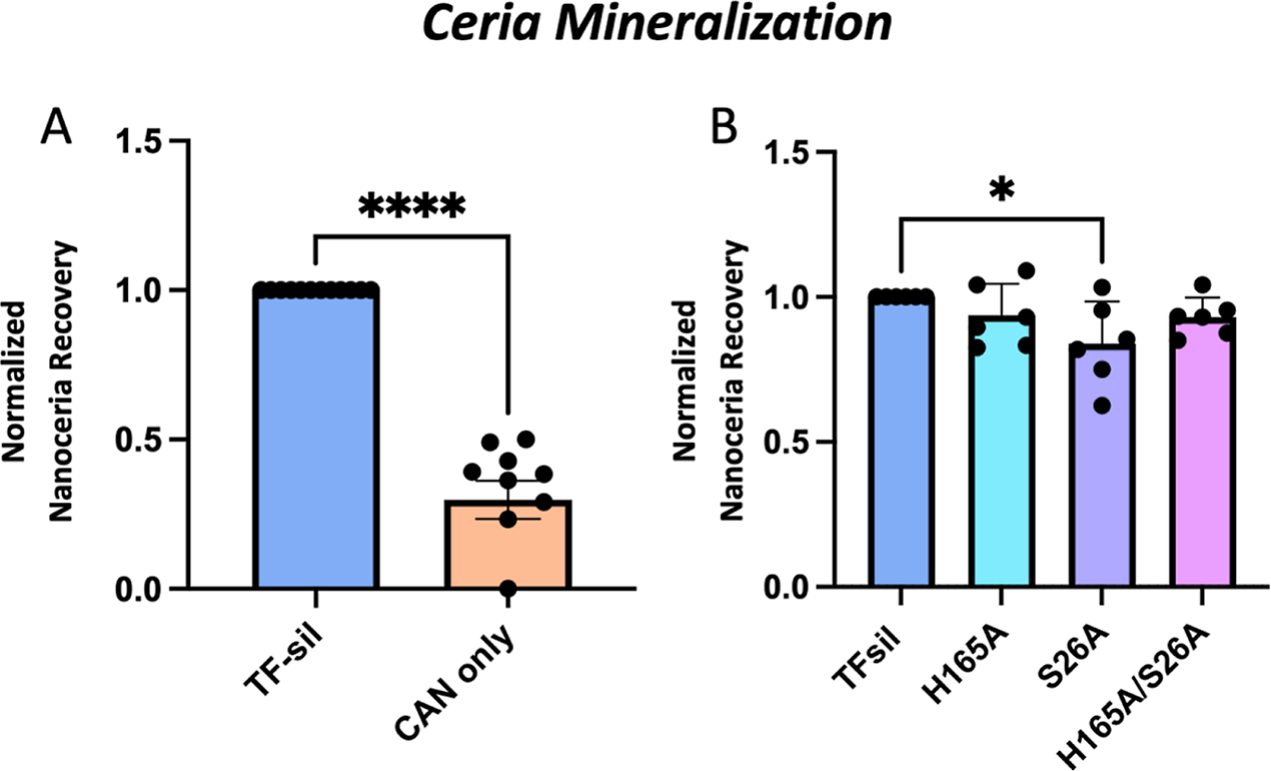
(A) Ceria mineralization for purified TF-silicatein in comparison to a ceria ammonium nitrate only, no protein control highlighting significantly greater nanoceria recovery with TF-silicatein than without. Paired, two-tailed *t*-test, *p* < 5 × 10^–6^, *n* = 10. (B) Ceria mineralization for purified TF-silicatein and TF-silicatein mutants H165A, S26A, and H165/S26A, showing a significant difference between TF-sil and S26A nanoceria recovery, but no significant difference with H165A and H165/S26A. One-way ANOVA *p* < 0.05, *n* = 6. Data originally quantified by moles, then percent recovery, and finally normalized to TF-silicatein outputs following the methods of Povarova et al. 2018. Further explanation is available in the Supporting Information.

Figure 1A illustrates the significant impact of TF-silicatein in ceria biomineralization as compared to a negative control condition where the protein was excluded (i.e., CAN only). This highlights the increased levels of ceric oxide production due to TF-silicatein as opposed to precursor autohydrolysis. A comparison of TF-silicatein with site-directed mutagenesis mutants H165A, S26A, and H165A/S26A shows no significant difference in normalized nanoceria recovery between TF-silicatein, H165A, and H165A/S26A. The mutant S26A, however, shows decreased normalized nanoceria recovery and therefore overall biomineralization activity compared to TF-silicatein (Figure 1B). These results align with the findings of Povarova et al. indicating that abolishing the putative catalytic triad residues results in minimal impact to overall biomineralization activity, therefore suggesting an alternative mechanism for silicatein biomineralization. Povarova et al. suggest templated biosilicification as a potential mechanism of silicatein activity; however, there are not any obvious domains for interrogation. Accordingly, we pursue a directed evolution approach with recombinant silicatein to probe the mechanism of biomineralization activity.

### Directed Evolution with Recombinant Silicatein

For directed evolution with recombinant silicate, we generated a random library via error-prone polymerase chain reaction (PCR) with dNTP analogues. Taking into account the length of silicatein, the dNTPs and dNTP analogues, as well as possibility of null mutations this library generated nearly 100,000 full-length mutants.^38^ As a preliminary screen for enhanced biomineralization activity, we challenged *Escherichia coli* survival with CAN, which is toxic to the cells. WT TF-silicatein rescues the cells from otherwise toxic concentrations of CAN, which established a threshold for sufficient biomineralization activity, presumably due to the sequestration and biomineralization of CAN to nanoceria (Figure S3). Accordingly, mutants that met or exceeded the WT survival threshold (2.5 mM CAN) were considered to have a promising biomineralization ability and further characterized. With CAN as precursor species, *E. coli* rely on silicatein to biomineralize this toxic compound to nontoxic ceric oxide, enabling *E. coli* survival.

### Select Silicatein Mutants Display Greater Biomineralization Activity

**Figure 2:**
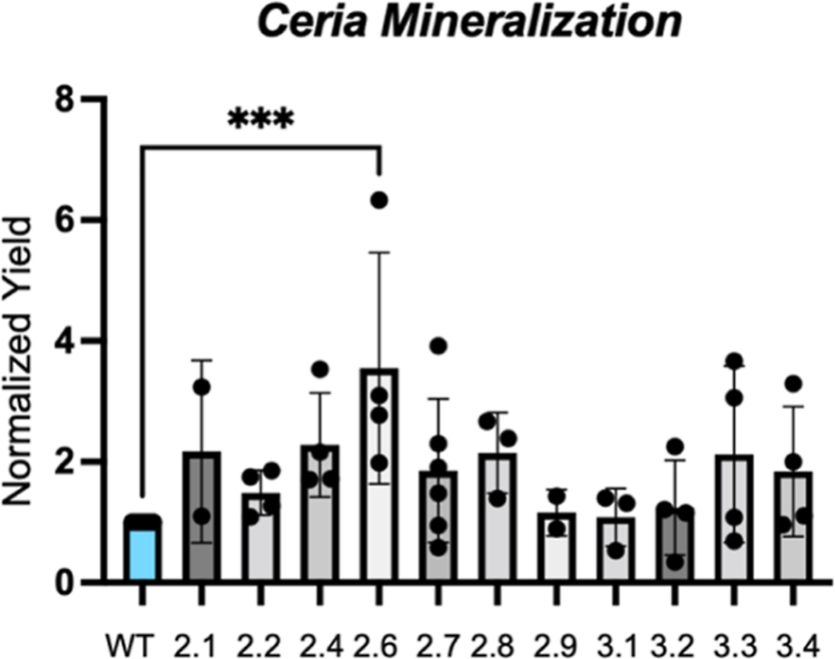
(A) Ceria mineralization of wild-type and mutant TF-silicateins in whole cell lysates, normalized to the production of WT. Each sample normalized by total protein concentration. Normalized nanoceria recovery calculated in the same way as shown in Figure 1. Two-way ANOVA, *n* > 2, ***P < 0.001.

The DNA of ten colonies who met the survival threshold of WT TF-silicatein was purified, retransformed, and overexpressed in *E. coli* for further characterization. Initial characterization of TF-silicatein mutants was determined via in vitro biomineralization activity associated with the whole cell lysate. For whole cell lysate screening assays, samples were normalized by the total protein concentration, as determined by a standard Bradford assay. In this way, the total protein concentration per sample remained consistent, although the silicatein concentration within the cell lysate and reaction mixture is expected to be variable. To evaluate biomineralization activity, whole cell lysate samples were incubated with CAN for 24 h and then nanoparticles were isolated via centrifugation. Total nanoceria biomineralization was quantified via an arsenazo III colorimetric assay (described in the Materials and Methods section) and yields were evaluated relative to the yields of WT protein, as described previously (Figure 2). Of these 10 preliminary mutants, mutant 2.6 exhibited significantly greater biomineralization yields compared to WT TF-silicatein, with average yields nearly 4× greater (Figure 2, Table S2).

**Figure 3:**
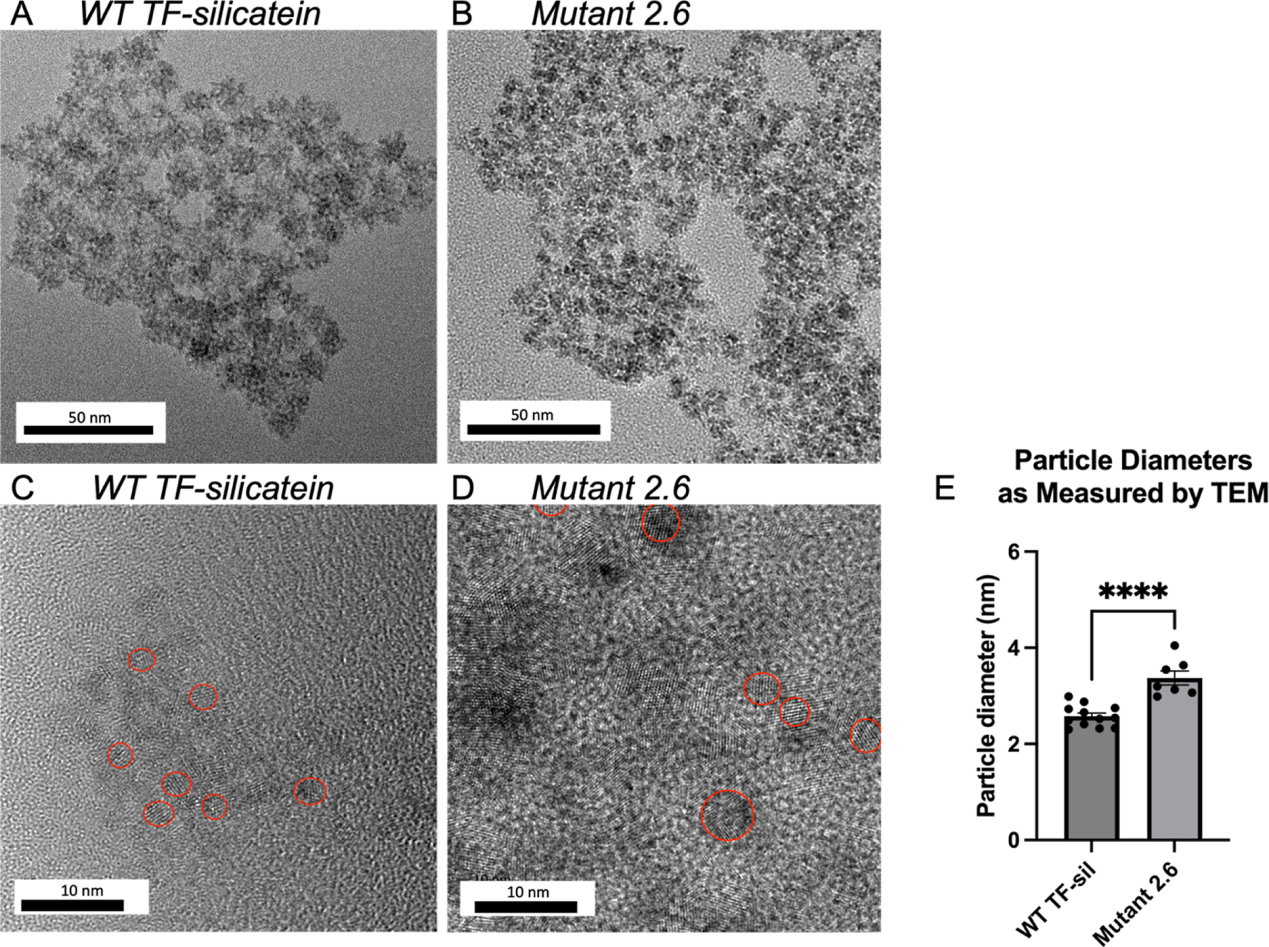
(A,C) Ceric oxide nanoparticles generated via in vitro biomineralization with WT TF-silicatein shown via TEM. Scale bars are 50 and 10 nm, respectively. Individual particles outlined with red. Average particle diameter 2.46 ± 0.17 nm. (B,D) Ceric oxide nanoparticles generated via in vitro biomineralization with mutant 2.6. Scale bars are 50 and 10 nm, respectively. Individual particles outlined with red. Average particle diameter 3.37 ± 0.15 nm. (E) A comparison of particle diameters, two-tailed unpaired *t*-test *p* = 0.0001, df = 17.

Preliminary transmission electron microscopy (TEM) examination of biomineralization products shows that the cell lysate of mutant 2.6 generates larger nanoparticles than WT TF-silicatein (Figure 3). Ceric oxide particles generated by mutant 2.6 measured an average of 3.37 ± 0.15 nm, while particles generated with WT TF-silicatein measured an average of 2.46 ± 0.17 nm. The 2.64 nm particles made with TF-silicatein are comparable to the 2.56 nm ceric oxide particles generated by Curran et al.^11^ The significant difference in the average particle size (*p* = 0.0001) may indicate that sequence changes in mutant 2.6 correspond to varied biomineralization activity. While there are many possible explanations for larger particle sizes, with consideration to the classical theory of crystal nucleation and growth, the larger particle size may be due to quicker nucleation and consumption of the monomer, resulting in size-broadening.^39^ Alternatively, the two-step mechanism of protein crystallization described by Gebauer and Cölfen suggests the formation of a dense fluid of prenucleation monomers that then undergoes a structural transition to crystalline particles.^40^ In this case, it might be theorized that mutant 2.6 enables sequestration of precursor and the formation of dense fluid prenucleation pockets that are physically larger than those formed by WT TF-silicatein, which may point to critical differences in the active site pocket of the protein. The PDB structure of silicatein with the earliest truncation seen in mutant 2.6 is shown in Figure S4. With consideration to nanoceria applications, the larger particles produced by mutant 2.6 may be more desirable for biomedical applications,^41^ while the 2 nm particles produced by WT TF-silicatein are particularly attractive for catalysis^11^ as the discrete size of nanoceria particles has a profound impact on surface chemistry.^42^

## Biomineralization Activity Associated with Mutants Corresponds with a Variety of Sequence Changes

DNA sequencing for TF-silicatein mutants revealed multiple different sequences associated with each mutant, consistent with multiple plasmids in each sample. In fact, chromatograms generated via Sanger sequencing were highly complex with an assortment of nucleotides identified at each position, making them difficult to interpret. These results were consistent with sequencing of “multiple vector transformants” as reported by Scanlon et al..^43−45^ To eliminate any possible contamination and limit complexity, the gene of interest was isolated via PCR from plasmid DNA and then sent for Sanger sequencing. Here, Sanger sequencing from the PCR of a given plasmid revealed a sequence different from a different PCR product of the same plasmid. These results are consistent with the presence of multiple plasmids in a single sample.

**Figure 4:**
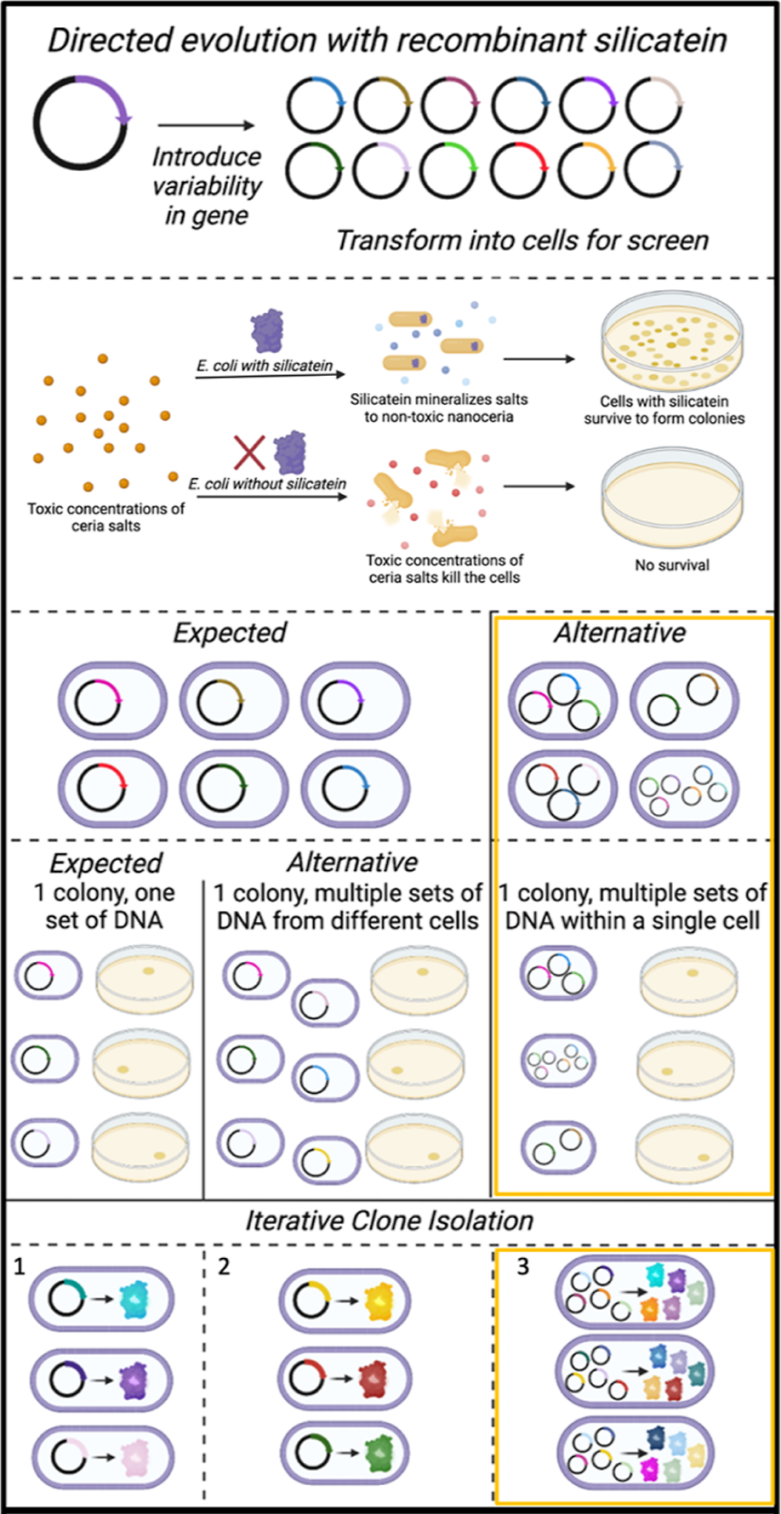
Expected and alternative outcomes of the experimental procedure surrounding directed evolution with recombinant silicatein are shown. The expected outcomes are consistent with traditional microbiology wisdom. Alternative scenarios have been documented in literature, however remain rare. The three potential outcomes following interative clone isolation are shown, with 1 and 2 being one set of DNA associated with each colony, and 3 (highlighted in yellow) showing multiple sets of DNA coinciding. Figure made with Biorender.

Previous work by Tomoiaga et al. 2022 illustrates the possibility and frequency for plasmid cotransformations.^46^ In cases of plasmid cotransformations, there are two possibilities: 1) one plasmid is transformed per cell; however, many cells containing different plasmids make up a single colony or 2) multiple plasmids are transformed per cell (these scenarios are outlined in Figure 4).^46^ Multiple plasmids coexpressed within a single cell are analogous with genetic chimerism and genetic mosaicism in nature.^47^ Furthermore, genetic mosaicism among marine diatoms, sponges, and corals has been widely documented.^47−53^ In fact, Rinkevich 2019 explores coral chimerism as a response to global warming,^52^ a selective pressure that could be considered analogous to our survival assay.

Similar challenges identifying a single DNA sequence for a given recombinant scenario have been documented previously by Heins et al. 2011 and Goldsmith et al. 2007, and are known to be more prevalent in cases of survival-based selection.^43,45^ Goldsmith et al. report techniques for separating plasmid cotransformations by repeated DNA purification, transformation, and clone isolation, which enable the separation of multiple plasmids within a colony as long as each plasmid is alone within the cell. However, our attempts in plasmid isolation, with at least five rounds of iterative clone isolation as described by Goldsmith et al. 2007, were unsuccessful. This inability to separate cotransformed plasmids is consistent with multiple plasmids present within the same cell (Figure 4).^46^ These results illustrate an artificial form of genetic mosaicism, where-in multiple copies of a single gene are different, and accordingly express different variations of the given protein.^47,51^

In the case of recombinant TF-silicatein, genetic mosaicism confers greater survival against CAN toxicity. Thus, multiple variants of each mutant protein are present in each colony, and therefore, multiple mutant proteins collaborate to achieve the biomineralization documented here. In addition, this suggests that silicatein is very permissive to a variety of sequence changes while maintaining catalytic activity as a multitude of mutants boosts biomineralization.

**Figure 5:**
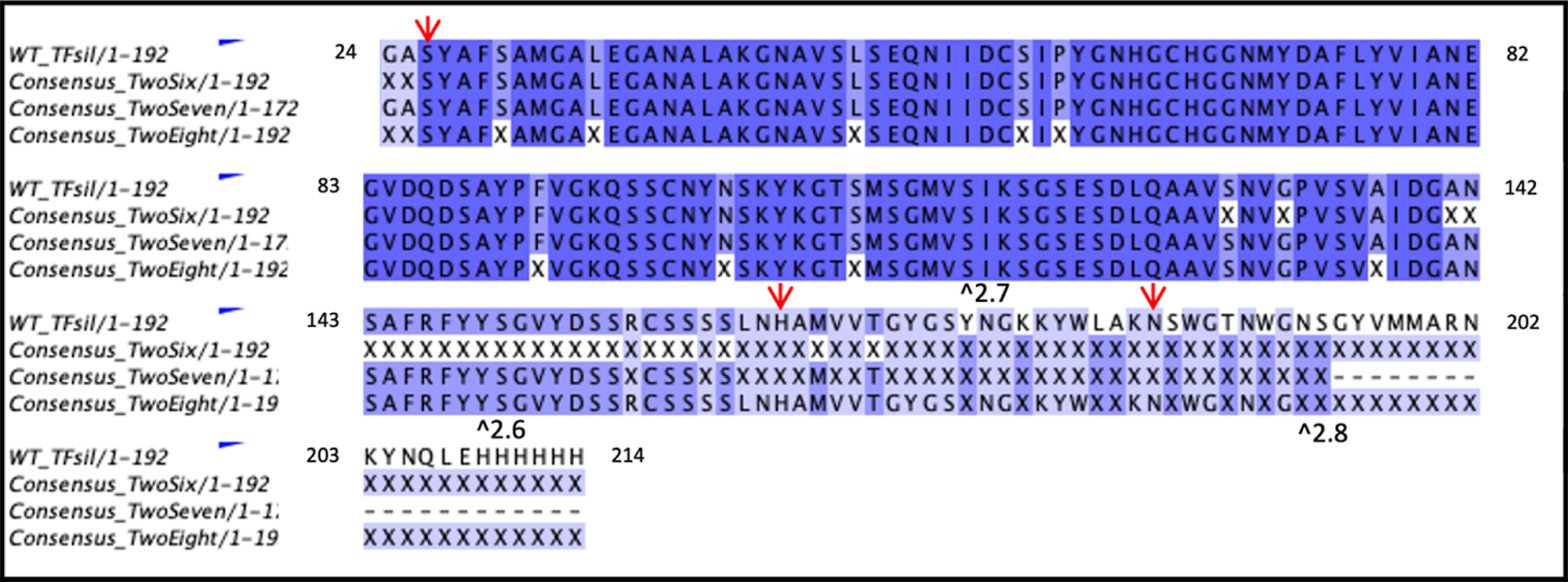
Sequence alignment for WT compared to >80% consensus sequences for 2.6, 2.7, and 2.8. Depth of coloration corresponds with percent identity. “X” refers to nonspecific amino acid. Catalytic triad residues are indicated with red arrows. The earliest stop codon for each consensus sequence are marked with “^” Sequence alignments prepared with EMBL-EBI 2022 and Jalview.

Utilizing the multiple sequences generated for each mutant (Figures S6–S8), we are able to establish consensus sequences (>80%) (Figure 5). The consensus sequences show limited conservation of the putative catalytic triad residues as well as many sequence truncations (Figure 5). Bawazer et al. 2012 reported a high amount of truncated proteins in their library sampling; however, here we are able to confirm that these truncated proteins retained biomineralization activity (Figure 2). Notably, some sequence truncations do abolish the catalytic triad residues, which suggests that an alternative mechanism supports biomineralization activity. It is likely that a combination of the catalytic triad, surface-templating,^28^ and additional processes combined lead to improvement in biomineralization observed. In light of these findings, further exploration into sequence truncations may be promising to interrogate silicatein’s mechanism of biomineralization.

With consideration to the significantly larger particle sizes produced by mutant 2.6, it is notable that most sequences generated for this mutant were truncated, with an 80% consensus maximum length of 171 amino acids (Table 1, Figure 5). These results may suggest that the N-terminal tail of silicatein has a role in biomineralization, directing the size of the particles produced. Alternatively, if a biomineralization mechanism based on intrinsic disorder and liquid–liquid phase separation within the protein directs biomineralization activity,^54^ it is possible that the loss of the N-terminal tail promotes intrinsic protein stability and folding. The estimated Instability Index of WT silicatein and mutant 2.6 (calculated via Expasy ProtParam) is shown in Table 1. The Instability Index accounts for known dipeptide combinations that result in unusually unstable proteins with an output score greater than 40 suggesting high instability.^55^ Mutants 2.6 b, c, f, g, and h each scored >40, suggesting high protein instability. This is relevant to the intrinsic disorder and liquid–liquid phase separation theory of biomineralization, as a more unstable protein could manifest in greater liquid–liquid phase separation, thus, promoting biomineralization in this way. Additionally, Table 1 shows the calculated isoelectric points for each 2.6 mutant, with all but one estimated to be acidic. Previous studies of biomineralization proteins in mollusks suggests that an acidic isoelectric point promotes biomineralization.^56,57^ With consideration to all of the above results, it is not possible to draw a single definitive conclusion between sequence changes and increased nanoparticle sizes generated by mutant 2.6; however, there are many possible contributing factors to the greater nanoceria biomineralization yields.

**Table 1:** Mutations and Truncations in Mutant 2.6^a^

	length (AA)	calculated PI	instability index	mutations
WT silicatein	191Begins at G24 with stop codon at 215	7.25	32.79	n/a
2.6a	170	5.49	33.53	G24L, Y27K, K178E, N193E, early stop codon at 194
2.6b	126	4.46	40.88	A42D, A142G, S143F, A144G, R146CEarly stop codon at 150
2.6c	160	5.83	41.91	Y180C, A183V, Stop codon at 184
2.6d	189	6.7	31.67	Y204X, Stop codon at 213
2.6e	172	6.31	39.1	Frameshift at AA 189, Stop codon at 196
2.6f	157	5.83	40.10	N176T, Stop codon at 181
2.6 g	190	**8.85**	**48.04**	Frame shift at AA 174, 39/40 remaining AA differ from WT. Stop codon at 214
2.6h	153	5.12	42.34	R157H, S161R, L163X, N164T H165Q, A166T, V168I, V169D, G171Q, Y172X, G173 K, Y175N,N176T, Stop codon at 177

a Please note, the numbering scheme of amino acid residues has been shifted to match those commonly associated with the catalytic triad. G1 in this work corresponds to G24 in the catalytic triad number system. As noted in Materials and Methods, all biomineralization experiments were conducted in 0.1 M Tris buffer, pH 7. Sequence alignments included in Supporting Information, Figures S6–S8.

Consensus sequences for 2.7 and 2.8 show mutations adjacent to the catalytic triad serine, which is similar to what Fairhead et al. 2008 showed via site-directed mutagenesis, postulating a larger space for precursor coordination and reaction.^33^ Interestingly, any substitution for G will result in some greater side chain, therefore increasing steric hindrance and limiting space around this S. Substitutions for A were frequently E, S, and C, which have been shown to coordinate with metals in biomineralization processes or contribute to the acid/base reaction themselves.^39,58,59^ As biomineralization activity is intact and enhanced, it is clear that silicatein tolerates a variety of sequence changes, and with the coexpression of multiple mutants, the *E. coli* selection host benefits from genetic mosaicism. An evaluation of silicatein from various sponge species, including *T. aurantia*, *S. domuncula*,^17^*P. ficiformis*,^18^*L. oparinae*,^19^*H. okadai*,^20^*A. queenslandica*,^21^ and even in freshwater sponge *L. baicalensis*(22) shows a minimum percent identity of 54% and a maximum percent identity of 73% (Figure S5, percent identifies calculated with NCBI MSA Viewer 1.25.0). With this in mind, it is unsurprising that the directed evolution of silicatein tolerates a multitude of sequence changes but maintains activity.

Studies of other marine organisms also reveal genetic mosaicism or varying genetic polymorphisms under selective pressure.^60−63^ Some theories suggest that this phenomenon is a result of cryptic genetic variation, which refers to variability within an organism’s genome that allows it to rapidly adapt to evolutionary pressures.^63^ In our work, silicatein is subjected to a novel selective pressure (CAN toxicity) and adapts by manifesting multiple variations of the silicatein mutant for the silicatein gene. Furthermore, studies of the enzyme malate dehydrogenase across a variety of marine species show individual amino acid changes in response to external pressure, with multiple individual sites contributing to enzymatic success and organism survival.^62^ Here, we effectively induce cryptic genetic variation in response to selective pressure via directed evolution, thus leading to genetic mosaicity. This highlights the importance of silicatein in marine sponge survival along with a propensity for broad sequence variability to achieve an improvement in biomineralization activity.

## Conclusions

An overall comparison of silicatein sequences from multiple sponge species highlights significant sequence variability, with percent identity ranging upward from 50%. Because the mutants generated in this work show functional production of ceria and sequence variability among sponge species is considerable, the sequence flexibility shown is consistent with prior observations regarding genetic variability among marine sponge species. No single optimally evolved silicatein mutant was identified in this work; the genetic mosaicism displayed suggests that multiple mutants coexisting confers augmented biomineralization activity. Additionally, genetic variability has been shown to increase marine sponge organism survival and adaptability in response to natural stressors and global warming.^60−63^ Furthermore, our study illustrates how directed evolution can reproduce genetic variability associated with selective pressure seen in nature, which may be a valuable tool for studying the organismal response to climate change.

These findings entail that in vitro biomineralization with silicatein for materials synthesis applications is unlikely to be optimized with a single version of silicatein and that the combination of multiple mutants, or an artificial genetic mosaicism, may be key for enhancing biomineralization. We show that truncated silicateiin mutants retain functionality, which may be leveraged for recombinant protein production: because the functional protein is smaller it is possible it will require decreased production time or have increased protein solubility. Furthermore, both site-directed mutagenesis with the catalytic triad and directed evolution results highlight that nanoceria production with silicatein is not solely mediated via a catalytic triad mechanism, thus directing focused efforts to maximize materials synthesis applications elsewhere.

In summary, this work shows the directed evolution of silicatein with a survival-based screening to highlight biomineralization activity. Interestingly, one individual “super silicatein” mutant has not emerged; rather, there is genetic mosaicism with several silicatein mutants leading to enhanced cell survival. In order to link a preferred phenotype to a defined genotype, each individual mutant should be produced and characterized individually. These experiments may provide critical insights into enabling future directed evolution and protein engineering projects.

## Materials and Methods

### Recombinant Protein Production and Purification

A pet28-TF-silicatein was transformed into BL21 *E. coli*. An individual colony was selected from a kanamycin LB-agar plate for a starter culture and then grown at 37 °C overnight shaking. Cells were separated via centrifugation at 3000*g* for 10 min then resuspended in the Terrific Broth and grown at 37 °C shaking for several hours with optical density at 600 nm measured periodically. At OD_600_ between 0.6 and 0.8, isopropyl-d-1-thiogalactopyranoside (IPTG) was added for a final concentration of 0.1 mM followed by overnight shaking incubation at 20 °C. Cells were recovered via centrifugation at 10,000*g* for 10 min and resuspended in lysis buffer (5% v/v 99% glycerol, 36 mM Tris HCl, 20 mM Tris Base, 300 mM sodium chloride, and 5 mM imidazole). The cells in lysis buffer were then incubated on ice for microtip sonication (Q500 QSonica) for alternating 20 s on/off cycles for 20 min and then centrifuged at 10,000*g* for 15 min. The supernatant was syringe-filtered and processed via immobilized metal affinity chromatography with Chelating Sepharose fast flow resin (ThermoFisher). The elution containing purified TF-silicatein was dialyzed at room temperature in 100 mM Tris buffer with 10,000 MWCO snakeskin dialysis tubing (ThermoFisher). Protein concentration was measured via A280 with a NanoDrop 1000 spectrophotometer and calculated with extinction coefficient 56,520 M^–1^ cm^–1^.”

### In Vitro Biomineralization

2 μM concentration of TF-silicatein fusion protein (or appropriate mutant), 2 mM CAN precursor, 100 mM Tris (pH 7), and deionized water were combined in a test tube at room temperature. Solutions were placed on an orbital mixer and incubated at room temperature for 24 h. Mineralized ceria nanoparticles were recovered via centrifugation at 20,000*g* for 30 min with Eppendorf 5424 centrifuge.

### Directed Evolution

#### Library Generation

Silicatein mutants were generated via error-prone PCR with dNTP analogues, adapted from Chao et al. 2006,^64^ and shown in Table 2.

**Table 2:** Conditions for EP-PCR Used in Library Generation

EP-PCR Part 1	
PCR reaction components	final concentration
10 × Taq buffer	1×
50 mM MgCl_2_	2 mM
10 μM forward primer	0.5 μM
10 μM reverse primer	0.5 μM
10 mM dNTPs	200 μM
template DNA	100 ng
20 μM 8-oxo-dGTP	2 μM
20 μM dPTP	2 μM
Fill to desired volume with DI H_2_O	
5 U/μL Taq DNA polymerase	0.05 U/μL

cycling conditions	
temperature (°C)	time
94	3 min
94	30 s
58	30 s
58	1 min
Repeat 30 cycles	
58	10 min

EP-PCR Part 2	
PCR reaction components	final concentration
10 × Taq buffer	1×
50 mM MgCl_2_	2 mM
10 μM forward primer	0.5 μM
10 μM reverse primer	0.5 μM
10 mM dNTPs	200 μM
extracted PCR product	minimum 10 ng
Fill to desired volume with DI H_2_O	
5 U/μL Taq DNA polymerase	0.05 U/μL

cycling conditions	
temperature (°C)	time
94	3 min
94	30 s
60	30 s
58	1 min
Repeat 30 cycles	
58	10 min

The forward primer for this random mutagenesis was: 5′ TAC ATA AGC TTG GTG CGA GTT ATG CTT TTT CTG C 3′ and the reverse primer was 5′ CCC ACC CTC GAG TTG ATT GTA TTT GTT 3′. This insert was then relegated into the pet28a(+) vector with the Trigger Factor (Accession # WP_096260434.1) according to the NEB T4 Ligation protocol. The ligation was transformed directly into BL21 *E. coli*, then screened as described below.

#### Survival Screening

The transformation culture was grown into saturation overnight in a 37 °C shaking incubator. Transformants were moved to the Terrific Broth and grown to an OD600 of 0.6 and then induced with IPTG overnight in a 20 °C shaking incubator. After induction, media was removed, cells were resuspended in water, then incubated in various concentrations of CAN, and allowed to grow overnight in a 37 °C shaking incubator. Cells were plated on LB agar according to CAN exposure concentration and grown at 37 °C for 18 h. Colonies grown at or above the critical concentration were selected for further screening. Figure S3 shows a representative image of survival screening. Cells from individual colonies were sampled, restreaked onto a new kanamycin LB-agar plate, and left to grow overnight at 37 °C. Cells from individual colonies were then sampled and inoculated in liquid media, grown overnight at 37 °C shaking, and then DNA was extracted and purified. This process was repeated at least five times for each mutant colony (i.e., five times for mutant colony 2.6, five times for mutant colony 2.7, etc.).

#### Secondary Screening

Following recombinant protein production and sonication, the total protein concentration for clarified cell lysate samples was normalized for the total protein concentration following quantitation with a Bradford assay. Lysates were then incubated with 2 mM CAN in 0.1 M Tris buffer (pH 7) for 24 h at room temperature. Mineralized product yields were measured via an arsenazo III colorimetric assay (described below).

### DNA Sequencing

Plasmid DNA of TF-silicatein mutants that were identified via secondary screening was amplified via PCR (Sil F primer 5′ TAC ATA AGC TTG GTG CGA GTT ATG CTT TTT CTG C 3′ and universal T7-term primer) and then PCR products were sent for Sanger sequencing with Eurofins Genomics. Sequence alignments were performed with Clustal Omega, EMBL-EBI tools,^65^ and JalView.

#### Arsenazo III Colorimetric Assay

Mineralized ceria was recovered via centrifugation at 20,000 × *g* for 30 min. The supernatant was discarded, and precipitates were dried and then resuspended in 10 mM citrate buffer (pH 3.0). A cerium ammonium nitrate standard curve in citrate buffer was prepared fresh for each reading. Samples were further diluted 1/15 in citrate buffer, then a final concentration of 2 mM arsenazo III dye was added, and adapted from Hogendoorn et al. 2018.^37^ Absorbance at 650 nm was read with a BioTek Synergy Neo2.

#### Transmission Electron Microscopy

Mineralized ceria from in vitro biomineralization was recovered via centrifugation at 20,000*g* for 30 min. The supernatant was discarded and precipitates dried and then resuspended in 10 mM citrate buffer (pH 3.0). 100 μL samples were applied to the TEM sample grid and let dry at room temperature. Samples were examined with an FEI Titan 80–300 transmission electron microscope. Image analysis and particle size measurement were conducted with ImageJ. To enhance contrast for particle measurements, over/under threshold was applied.

## Data Availability

The data sets generated and/or analyzed during the current study are available in the GenBank BankIt repository (submission IDs 2809913, 2809920, 2809923).

## Supporting Information Available

Coding region for TF-silicatein pet28(a)+ plasmid, Arsenazo III colorimetric assay: standard curve and data analysis example, nanoceria yields for WT TF-silicatein and mutants in cell lysate, ceria mineralization: comparing TF-silicatein and Cathepsin L, survival assay: BL21 *E. coli* with ceria ammonium nitrate, sequence alignment for silicatein in different sponges, sequence alignment for mutant 2.6, sequence alignment for mutant 2.7, and sequence alignment for mutant 2.8 (PDF)
