# Supplementary material for: Directed Evolution of Silicatein Reveals Biomineralization Synergism between Protein Sequences
Source: ACS Omega. 2025 Jan 6;10(1):334–43. doi: 10.1021/acsomega.4c06359 (PMC11740617; doi:10.1021/acsomega.4c06359)
Supplement: Supplementary file 1 — ao4c06359_si_001.pdf [file ao4c06359_si_001.pdf]

Directed Evolution of Silicatein Reveals Biomineralization Synergism Between Protein Sequences

Authors: Toriana N Vigil[1], Mary-Jean C. Rowson[2], Abigail J. Frost[1], Abigail R. Janiga[1], Bryan W. Berger[1,2]*

1. Department of Chemical Engineering, University of Virginia
2. Department of Biomedical Engineering, University of Virginia

Table of Contents	
	Page number
**Table S1.** Coding region for TF-silicatein pet28(a)+ plasmid	S2
**Figure S1**. Arsenazo III Colorimetric Assay: Standard Curve and Data Analysis Example<br>• **Figure S1. A.** Standard curve for Arsenazo 3<br>• **Figure S1. B.** Method for converting calculated moles to percent recovery<br>• **Figure S1. C.** Method for normalizing percent recovery to the percent recovery of WT TF-sil	S2-S4
**Table S2.** Nanoceria Yields for WT TF-silicatein and Mutants in Cell Lysate	S5
**Figure S2.** Ceria Mineralization: Comparing TF-silicatein and Cathepsin L	S6
**Figure S3.** Survival Assay: BL21 *E. coli* with Ceria Ammonium Nitrate	S7
**Figure S4.** PDB structure 6ZQ3 with Earliest Truncation in Mutant 2.6b	S8
**Figure S5.** Sequence Alignment for Silicatein in Different Sponges	S8
**Figure S6.** Sequence Alignment for Mutant 2.6	S9
**Figure S7.** Sequence Alignment for Mutant 2.7	S10
**Figure S8.** Sequence Alignment for Mutant 2.8	S11

Supplemental Information

Table S1. Coding region for TF-silicatein in the pet28a(+) plasmid

Coding region for TF-silicatein in the pet28a(+) plasmid
MGSSHHHHHH SSGLVPRGSH MASMTGGQQM GRGSMQVSVE TTQGLGRRVT ITIAADSIET AVKSELVNVA KKVRIDGFRK GKVPMNIVAQ RYGASVRQDV LGDLMSRNFI DAIIKEKINP AGAPTYVPGE YKLGEDFTYS VEFEVYPEVE LQGLEAIEVE KPIVEVTDAD VDGMLDTLRK QQATWKEKDG AVEAEDRVTI DFTGSVDGEE FEGGKAPDFV LAMGQGRMIP GFEDGIKGHK AGEEFTIDVT FPEEYHAENL KGKAAKFAIN LKKVEERELP ELTAEFIKRF GVEDGSVEGL RAEVRKNMER ELKSAIRNRV KSQAIEGLVK ANDIDVPAAL IDSEIDVLRR QAAQRFGGNE KQALELPREL FEEQAKRRVV VGLLLGEVIR TNELKADEER VKGLIEEMAS AYEDPKEVIE FYSKNKELMD NMRNVALEEQ AVEAVLAKAK VTEKETTFNE LMNQQALEVL FQGPLVPRGS IEGREAAAKE AAAKGASYAF SAMGALEGAN ALAKGNAVSL SEQNIIDCSI PYGNHGCHGG NMYDAFLYVI ANEGVDQDSA YPFVGKQSSC NYNSKYKGTS MSGMVSIKSG SESDLQAAVS NVGPVSVAID GANSAFRFYY SGVYDSSRCS SSSLNHAMVV TGYGSYNGKK YWLAKNSWGT NWGNSGYVMM ARNKYNQLEH HHHHH*

Figure S1. Arsenazo III Colorimetric Assay: Standard Curve and Data Analysis Example

Please note that this data set is one of ten total data sets included in analysis for this work.

A cerium ammonium nitrate standard curve in 10 mM citrate buffer (pH 3.0) was prepared fresh for each reading. Standard curve ranged from 1.25 x 10^{-7} moles to 0 moles ceria and a final concentration of 2 mM arsenazo III dye was added, adapted from Hogendoorn, *et al.* 2018.[22] Absorbance at 650 nm was read with a BioTek Synergy Neo2.

Figure S1. A. Standard curve for Arsenazo 3

Standard Curve			
Moles (Ceria)	Absorbance 1	Absorbance 2	Absorbance 3
6.25E-08	---	1.78	1.425
3.125E-08	---	1.023	0.867
1.5625E-08	0.774	0.689	0.62
0	0.416	0.441	0.476

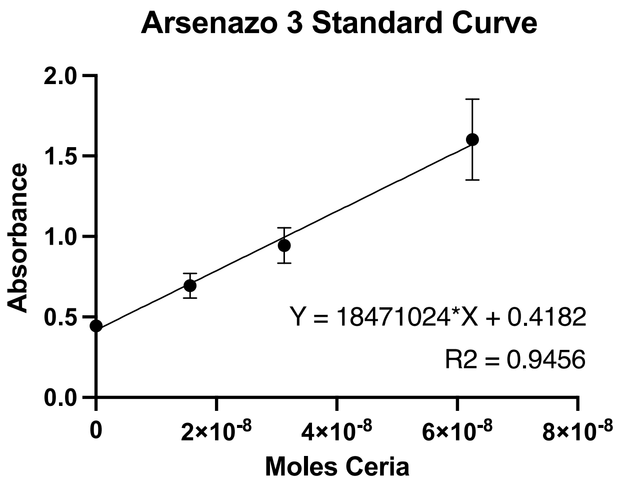

Mineralized ceria samples were resuspended in 10 mM citrate buffer (pH 3.0). Samples were further diluted 1/15 in citrate buffer and a final concentration of 2 mM arsenazo III dye was added. Absorbance at 650 nm was read with a BioTek Synergy Neo2.

Utilizing best-fit line from standard curve in Figure S1.A, absorbance values were converted to moles, which were then transformed into "percent recovery" with consideration to total possible ceria concentration of $1x10^{-7}$ moles.

Figure S1. B. Method for converting calculated moles to percent recovery

Sample	Absorbance			Calculated (moles) Y = 18471024*x + 0.4182			Average (moles)	Percent recovery
2.2	1.289	1.339	1.304	4.714E-08	4.985E-08	4.795E-08	4.831E-08	48%
2.4	2.129	2.196	2.183	9.262E-08	9.624E-08	9.554E-08	9.480E-08	94%
2.6	2.635	2.655	2.679	1.200E-07	1.211E-07	1.224E-07	1.211E-07	121%
2.7	0.954	0.898	0.861	2.900E-08	2.597E-08	2.397E-08	2.631E-08	26%
2.8	2.281	2.43	2.33	1.008E-07	1.089E-07	1.035E-07	1.044E-07	104%
WT	1.224	1.084	1.115	4.362E-08	3.604E-08	3.772E-08	3.913E-08	39%

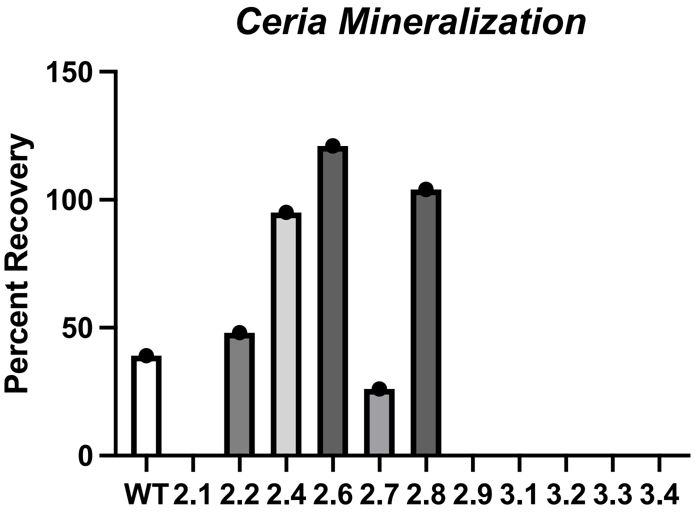

Finally, percent recovery values were transformed to a normalized yield based on WT TF-sil following the methods of Povarova, *et al.* 2018.

Figure S1. C. Method for normalizing percent recovery to the percent recovery of WT TF-sil.

	Calculated Percent Recovery	Normalized to WT TF-sil
WT	39%	1
2.2	48%	1.1
2.4	94%	2.2
2.6	121%	2.8
2.7	26%	0.6
2.8	104%	2.4

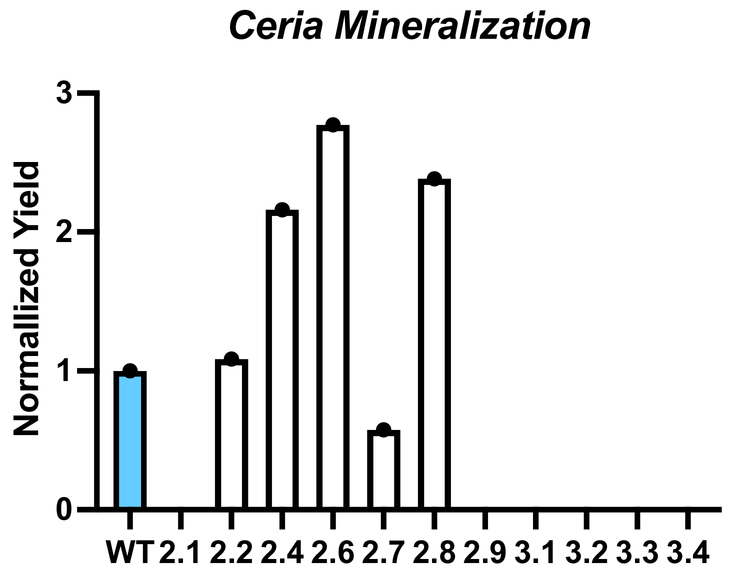

Table S2. Normalized nanoceria yields for WT and mutant biomineralization assays with cell lysates. In order to simplify comparisons and account for trial-to-trial variability, the nanoceria yields for WT protein were normalized to 1. For each biomineralization assay with a mutant, the WT was made and tested in tandem.

	n	Mean	95% Confidence Interval	Std Dev	SEM
WT	14	1.00*	---	----	----
2.1	2	2.17	-11.4 – 15.7	1.51	1.07
2.2	4	1.49	0.90 - 2.08	0.370	0.185
2.4	4	2.28	0.91 – 3.65	0.859	0.430
2.6	4	3.55	0.498 – 6.59	1.92	0.958
2.7	6	1.85	0.61 – 3.10	1.19	0.485
2.8	3	2.15	0.49 – 3.81	0.39	0.49
2.9	2	1.16	- 2.27 – 4.59	0.38	0.27
3.1	3	1.08	- 0.11 – 2.27	0.48	0.28
3.2	4	1.24	- 0.01 – 2.48	0.78	0.39
3.3	4	2.12	- 0.20 – 4.45	1.46	0.73
3.4	4	1.84	0.130 – 3.55	1.07	0.537

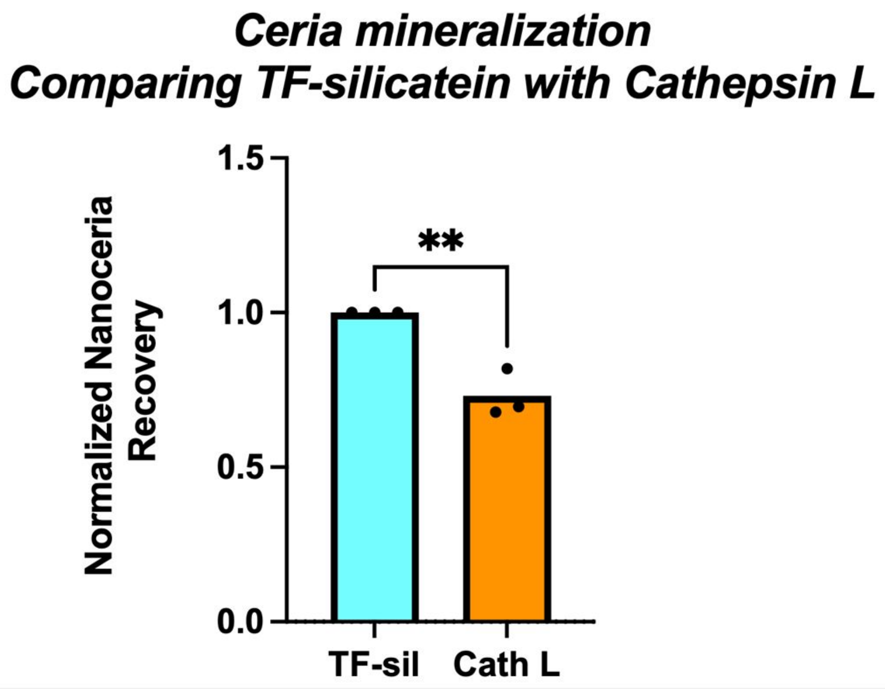

Figure S2. Comparing ceria mineralization with hexa-histidine tagged TF-silicatein and hexa-histidine tagged Cathepsin L. Unpaired two-tailed t-test, ** $p<0.005$. Normalized nanoceria recovery calculated in the same way as Figure 1. This result shows that biomineralization yields due to presence of hexa-histidine tags associated with an arbitrary protein is significantly less than that of TF-silicatein, highlighting the role of the enzyme in biomineralization activity.

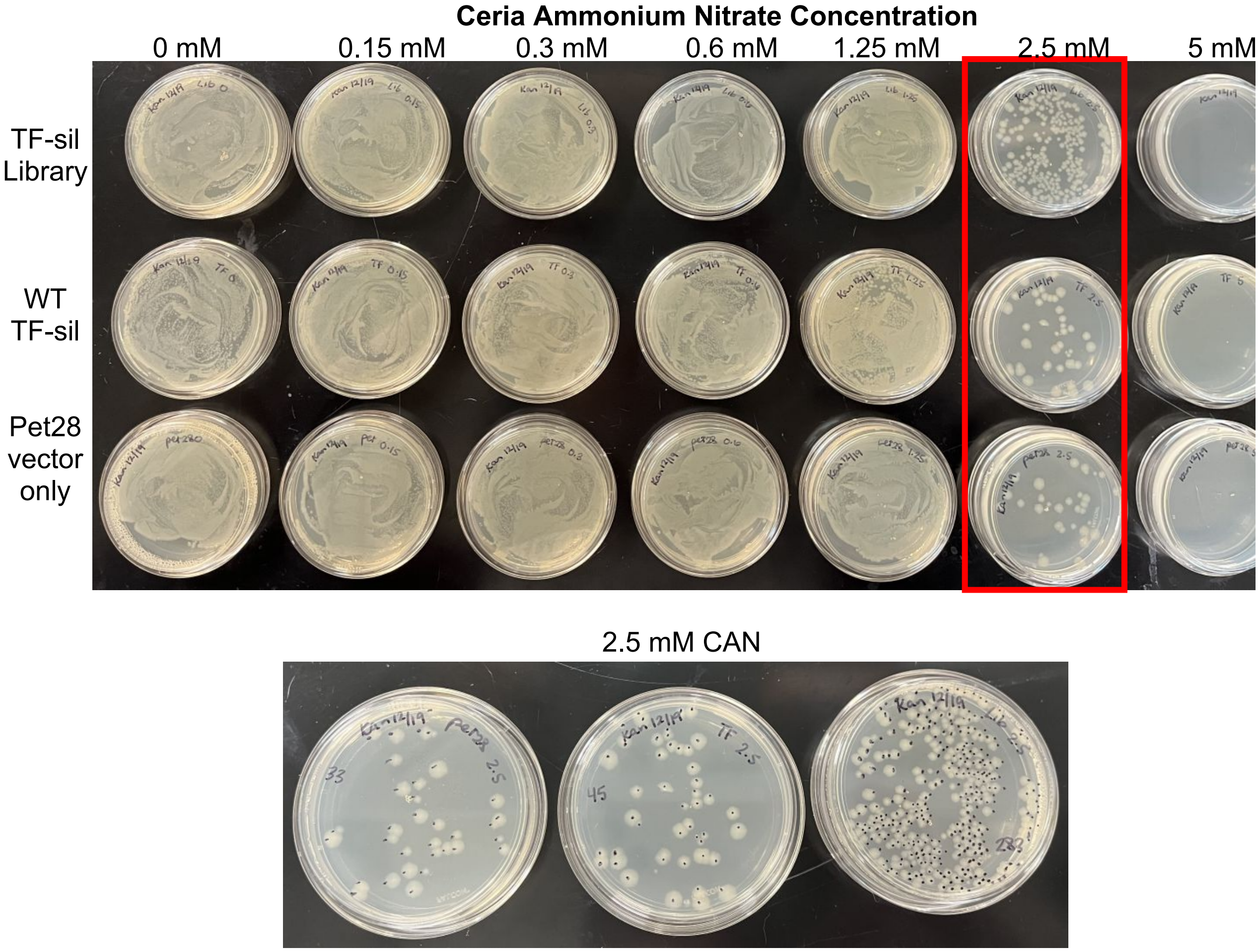

Figure S3. Establishing toxic concentration of ceria ammonium nitrate. *E. coli* with pet28 vector only, WT TF-silicatein, and TF-silicatein library. Notable differences in *E. coli* survival occur at 2.5 mM ceria ammonium nitrate, boxed in red. A closer view of 2.5 mM ceria ammonium nitrate is shown, with 33, 45, and 288 colonies at pet28 vector only, WT TF-silicatein, and TF-silicatein library respectively.

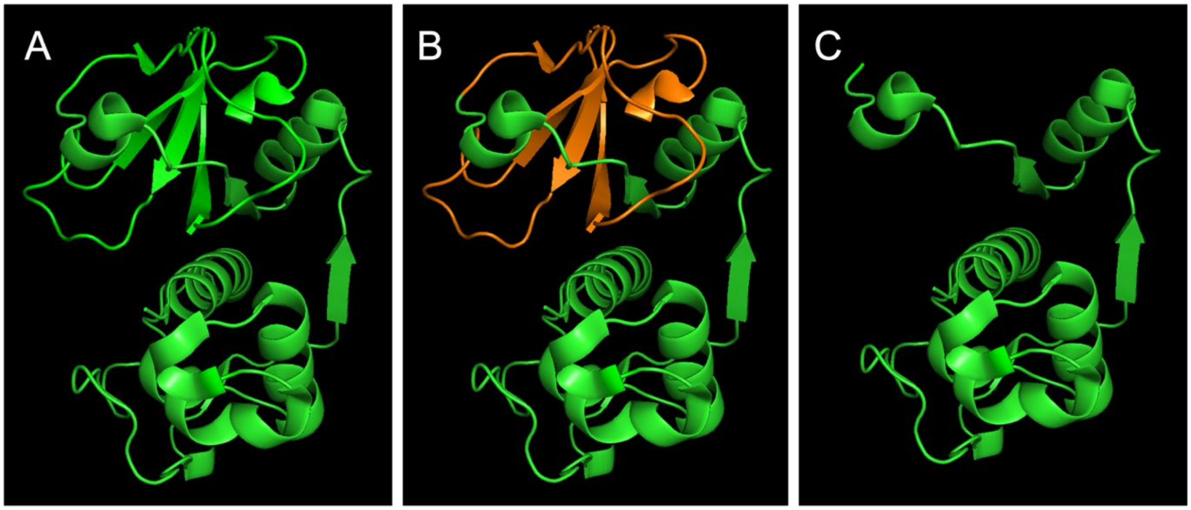

Figure S4. Silicatein structure from PDB 6zQ3. A) Full length silicatein from WT TF-sil in green, B) Full length silicatein from WT TF-sil. Orange color highlights AA from earliest truncation of mutant 2.6b to end of protein. C) Silicatein as it might appear in mutant 2.6b. Figure made with Pymol.

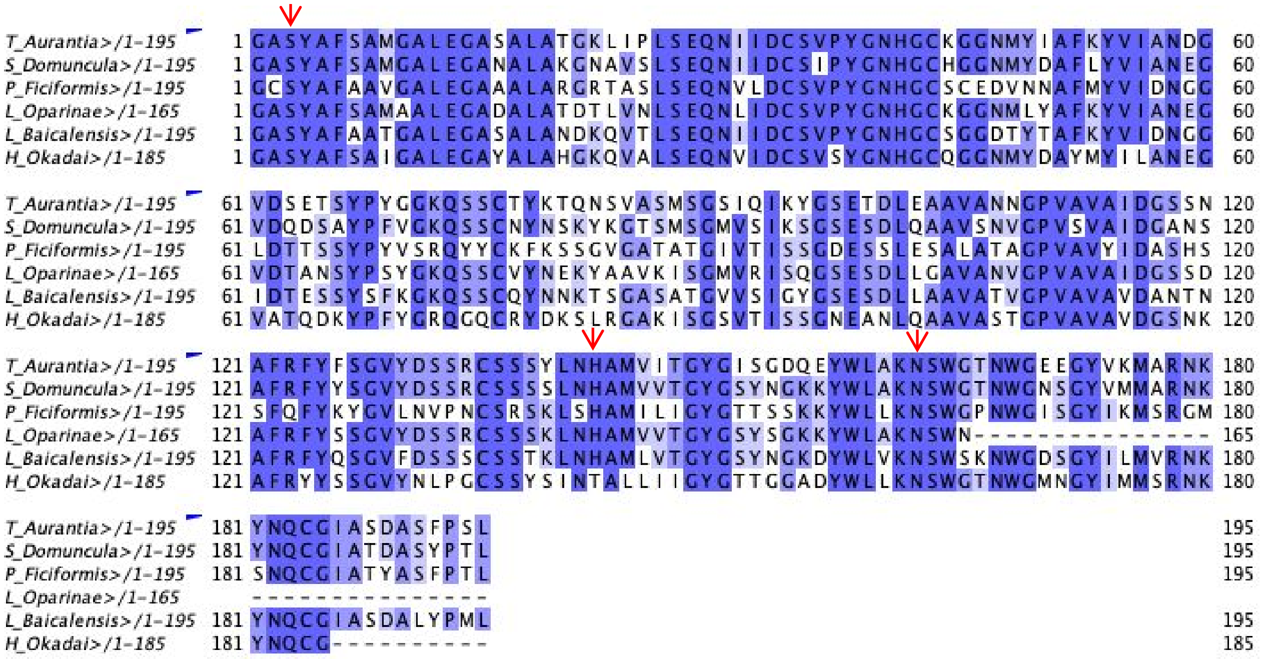

Figure S5. Sequence alignment for silicatein found in *T. aurantia* compared to other sponges. Depth of coloration corresponds with percent identity. Catalytic triad residues are indicated with red arrows. Sequence alignments prepared with EMBL-EBI 2022 (Ref 45) and Jalview.

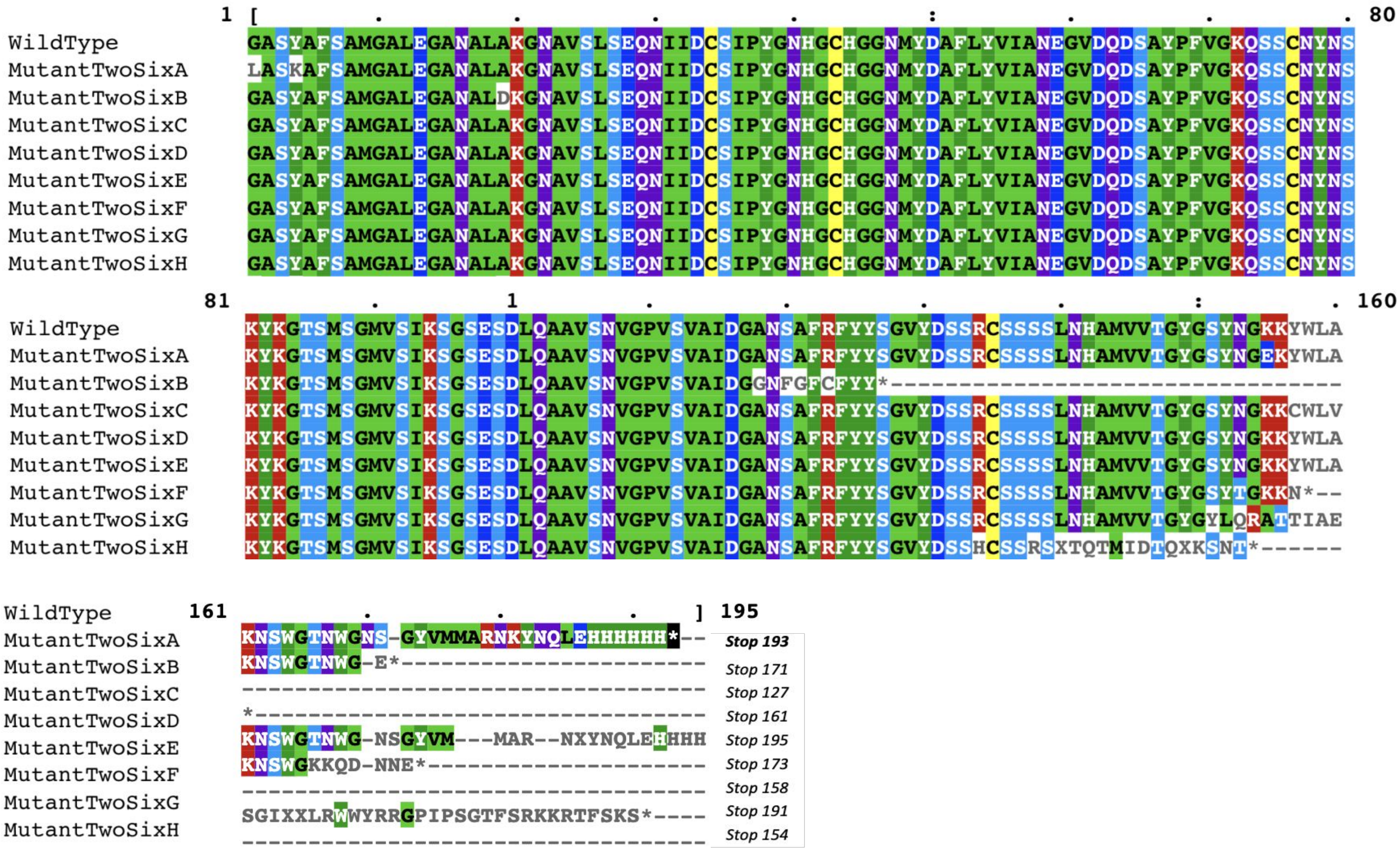

Figure S6. Sequence alignment for mutant 2.6, showing unique sequences identified with each sequencing trial as compared to the WT TF-silicatein sequence. Residues are colored based on amino acid properties according to EMBL-EBI classifications. Location of sequence Sequence alignments prepared with EMBL-EBI 2022.

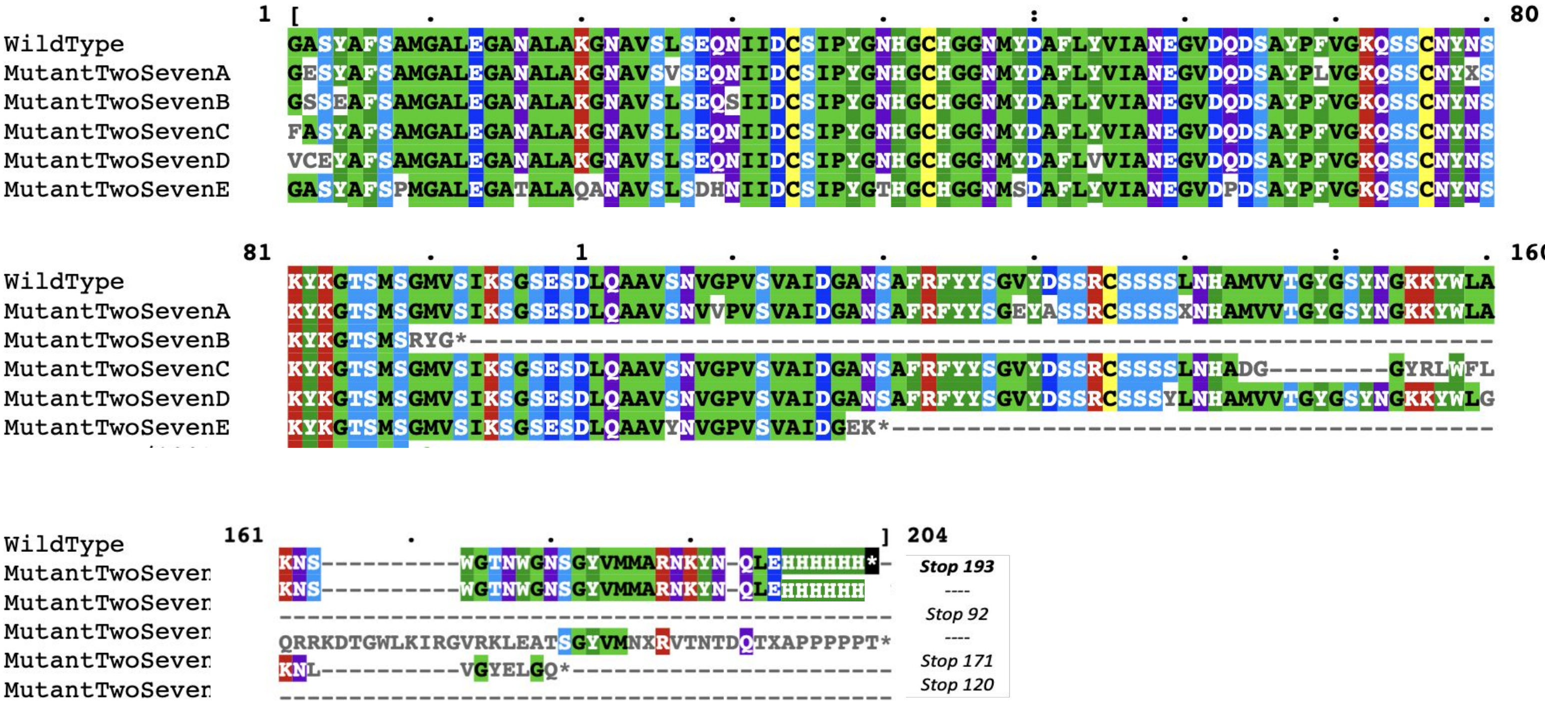

Figure S7. Sequence alignment for mutant 2.7, showing unique sequences identified with each sequencing trial as compared to the WT TF-silicatein sequence. Residues are colored based on amino acid properties according to EMBL-EBI classifications. Location of sequence Sequence alignments prepared with EMBL-EBI 2022.

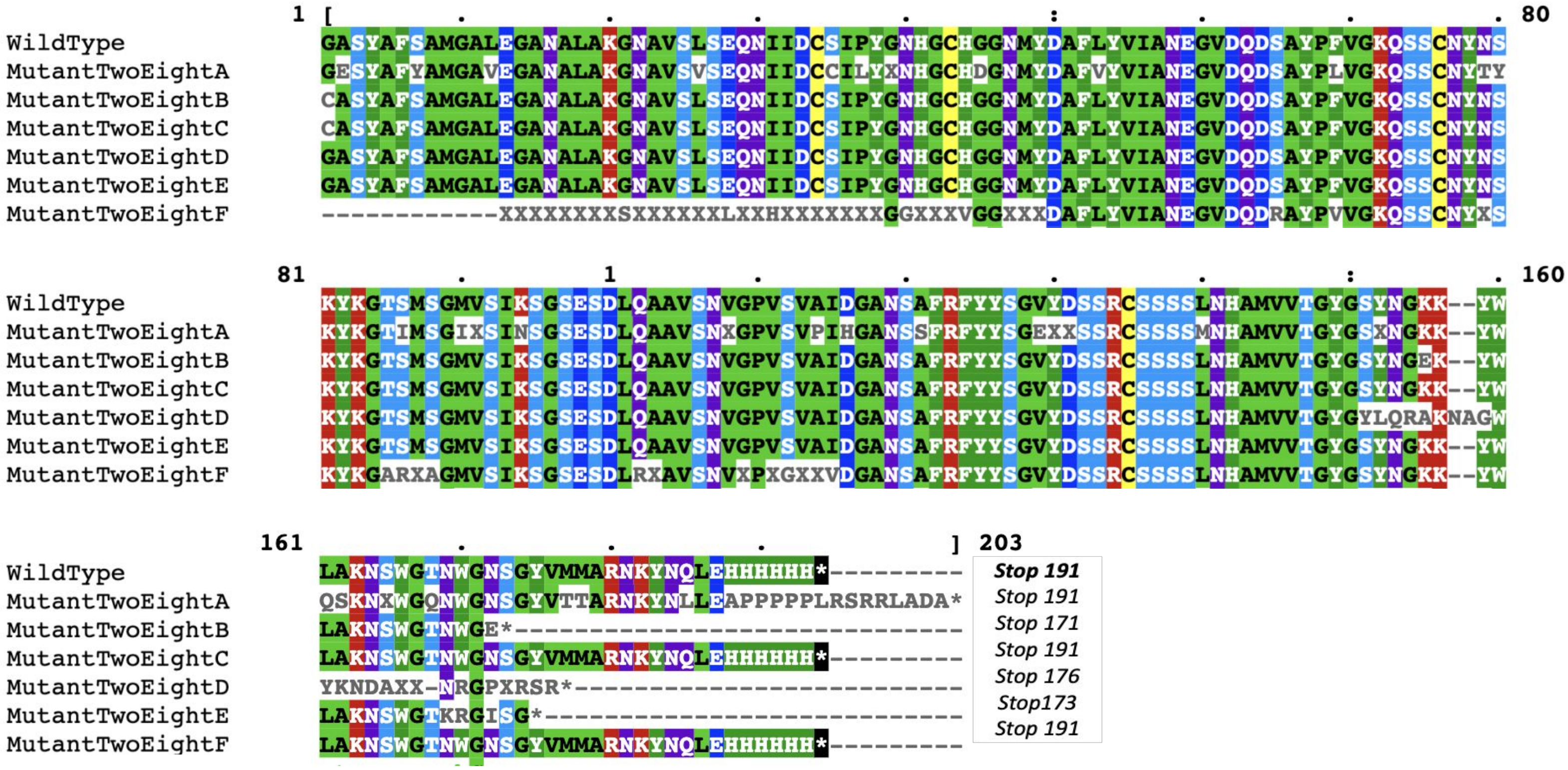

Figure S8. Sequence alignment for mutant 2.8, showing unique sequences identified with each sequencing trial as compared to the WT TF-silicatein sequence. Residues are colored based on amino acid properties according to EMBL-EBI classifications. Location of sequence Sequence alignments prepared with EMBL-EBI 2022.
